# Learning Auditory Space: Generalization and Long-Term Effects

**DOI:** 10.1371/journal.pone.0077900

**Published:** 2013-10-22

**Authors:** Catarina Mendonça, Guilherme Campos, Paulo Dias, Jorge A. Santos

**Affiliations:** 1 Cluster of Excellence Hearing4all, Carl Von Ossietzky University of Oldenburg, Oldenburg, Germany; 2 Department of Signal Processing and Acoustics, Aalto University, Espoo, Finland; 3 Centro Algoritmi, School of Engineering, University of Minho, Guimarães, Portugal; 4 Institute of Electronics and Telematics Engineering, University of Aveiro, Aveiro, Portugal; 5 Department of Basic Psychology, University of Minho, Braga, Portugal; University of Salamanca- Institute for Neuroscience of Castille and Leon and Medical School, Spain

## Abstract

**Background:**

Previous findings have shown that humans can learn to localize with altered auditory space cues. Here we analyze such learning processes and their effects up to one month on both localization accuracy and sound externalization. Subjects were trained and retested, focusing on the effects of stimulus type in learning, stimulus type in localization, stimulus position, previous experience, externalization levels, and time.

**Method:**

We trained listeners in azimuth and elevation discrimination in two experiments. Half participated in the azimuth experiment first and half in the elevation first. In each experiment, half were trained in speech sounds and half in white noise. Retests were performed at several time intervals: just after training and one hour, one day, one week and one month later. In a control condition, we tested the effect of systematic retesting over time with post-tests only after training and either one day, one week, or one month later.

**Results:**

With training all participants lowered their localization errors. This benefit was still present one month after training. Participants were more accurate in the second training phase, revealing an effect of previous experience on a different task. Training with white noise led to better results than training with speech sounds. Moreover, the training benefit generalized to untrained stimulus-position pairs. Throughout the post-tests externalization levels increased. In the control condition the long-term localization improvement was not lower without additional contact with the trained sounds, but externalization levels were lower.

**Conclusion:**

Our findings suggest that humans adapt easily to altered auditory space cues and that such adaptation spreads to untrained positions and sound types. We propose that such learning depends on all available cues, but each cue type might be learned and retrieved differently. The process of localization learning is global, not limited to stimulus-position pairs, and it differs from externalization processes.

## Introduction

Over the last decades, many scientific advances have demonstrated the neural plasticity and experience-based shaping of brain processes. Relearning auditory space from new head-related auditory cues is one of such cases. To be able to localize sounds, one must learn each sound position cue, which is always shaped by one’s own head and torso, and successively recalibrate to it through feedback. Alterations to auditory space cues can take place as the head size changes with age, through surgical means, with cochlear implants or hearing aids, or when audition declines with aging [1,2]. Analyzing how humans learn to localize with altered auditory cues will bring new insights on how the auditory space representations are formed and adjusted throughout life, with potential applications to hearing rehabilitation and auditory assistive technologies [3–5].

Several studies have revealed that animals learn to localize from altered sound cues. King and collaborators [1] recorded responses of neurons in the ferret’s primary auditory cortex to individualized and non-individualized virtual sound sources. They found that the structure of the spatial response fields changed significantly when non-individualized sounds were presented. But, through intensive training, ferrets relearn to localize sounds with altered cues [6,7]. In humans, altering both ears with molds immediately impairs elevation localization. Elevation localization is mostly affected by cues provided by the spectral shaping of the head and pinnae, rather than from the cues due to differences in sound signals arriving at both ears. Changing the shape of the pinnae dramatically affects such spectral cues, thus impairing elevation localization. But by wearing such molds for several weeks, accurate performance is steadily reacquired [8]. Interestingly, when the subjects take the molds off, their elevation localization accuracy remains unchanged, despite having been trained in the new cues. Irving and Moore [9] trained sound localization in humans hearing with and without a unilateral earplug. With the plug inserted in one ear the azimuth localization was impaired, due to its strong dependence upon binaural cues, namely the difference in time and level of the sounds arriving at both ears. Over 5 days wearing the plug, azimuth localization continuously improved and, upon plug removal, azimuth localization was again at the pre-plug accuracy levels. 

Studies on human auditory space adaptations have often been conducted with head-related filters. Those filters can be measured as a binaural impulse response for each source position, known as *Head Related Impulse Response* (*HRIR*), or by its Fourier transform, the Head Related Transfer Function (HRTF). With those filters, it is possible to reproduce through headphones the sound as it would be heard by a given person or model. It is also possible to present the sound filtered by someone else’s HRTFs, or any combination of these filters. Wright and Zhang [10] reviewed the literature on auditory localization learning with normal/individualized and altered/non-individualized cues in human adults. With altered cues, partial adaptation has been reported with a variety of cue manipulations, such as altered interaural time difference. For normal/individualized cues, significant improvement has only rarely been observed. These findings point out that improving localization accuracy with one’s own unaltered ears might not always occur, mostly because one might already have reached the optimal localization performance; but when altered sounds are trained, learning might take place because the initial performance is low.

To explore such learning potential, some training approaches have been proposed to teach humans how to localize with non-individualized head-related cues [11,12]. These training approaches used highly complex real-time virtualization systems that took advantage of the subject’s own head movement and therefore provided vestibular and proprioceptive cues, coupled with the audiovisual feedback of the virtual environment. After several days of training, subjects improved sound localization accuracy and this improvement lasted up to one month [11] and seemed to generalize to other untrained positions [12].

In a previous study [13] we addressed the learning of auditory localization with non-individualized head-related transfer functions in a simple setting. Using only passive contact to static free-field sounds without head motion or feedback did not result in any improvement in azimuth localization accuracy. On the other hand, a short training program, of less than one hour, involving active learning and response feedback, significantly improved the localization in both azimuth and elevation. These results revealed that auditory space learning might occur much faster than previously thought, and under much simpler conditions. Interestingly, both in azimuth and in elevation, listeners were trained in only 3 or 5 source positions, but improvement was found for all in-between source positions in the after test, suggesting some spatial generalization. 

Some spatial learning generalization effects have been suggested before [10], but they have never thoroughly been analyzed or directly tested. Understanding such effects might cast new insights on how the auditory spatial maps are formed and recalibrated, how the brain learns to associate the sound cues to positions in space, and how this association is represented. We designed a longitudinal learning study to assess the effects of time, stimulus type, previous experience, and sound source position. We also assessed the quality of the spatial sound experience by addressing externalization levels. Non-individualized sounds are often felt as less spatial or less compelling, typically being reported as felt inside the head or between ears, rather than externalized. This additional measure allowed us to better understand the quality of the training process. 

## Methods

### Overview

This study comprised several successive tests. In the main condition, there was an elevation training experiment and an azimuth training experiment. Each of them integrated a pre-test, a training session, and five post-tests. In the control condition there were also elevation and azimuth experiments, but they only comprised the pre-test, training, and two post-tests.

### Participants

Twelve inexperienced subjects, aged 20 to 55 (33 on average), participated in the main condition experiments. Due to technical issues, namely a large number of missing records in some sessions, only 10 subjects (20 to 55 – average 34) were considered in the data analysis. The control condition experiments involved nine subjects (19 to 22 - average 20), divided into three equal-sized groups.

All participants had normal hearing, checked by standard audiometric screening at 500, 750, 1000, 1500 and 2000 Hz, with auditory thresholds below 15 dB HL, and none showed interaural sensitivity differences above 5 dB HL. All the experiments were conducted in accordance with the Declaration of Helsinki and the resulting data were processed anonymously. 

### Ethics statement

All the participants were informed about the purpose of the experiments and provided written consent. The experiment was approved by the ethics committee of the School of Psychology, University of Minho. The experiment was conducted in accordance with the principles stated in the 1964 Declaration of Helsinki.

### Stimuli

The experimental stimuli were based on anechoic recordings of speech (Portuguese word “atum” (tuna)), and on computer-generated white noise, both with the duration of 3s. These sound files were convolved with head-related impulse response pairs corresponding to the simulated source position. The HRIR set used, taken from the CIPIC database [14], was measured on a manikin with constant distance of 1m between the sound source and the center of the manikin’s head. The actual stimuli, reproduced with a Realtec Intel 8280 IBA sound card, were presented through a set of Etymotics ER-4B MicroPro flat-response ‘in-ear’ earphones.

For the azimuth localization tests, ten source positions were considered in the horizontal plane (i.e. at constant 0° elevation), with azimuth ranging from front to right at 10° intervals: 0° (front), 10°, 20°, 30°, 40°, 50°, 60°, 70°, 80°, and 90° (right).

The elevation localization training tests used the same number of virtual source positions, now varying in elevation on the median plane (fixed 0° azimuth) with the same 10° angular spacing: 0° (front), 10°, 20°, 30°, 40°, 50°, 60°, 70°, 80°, and 90° (head top).

### Procedure

**Figure 1:**
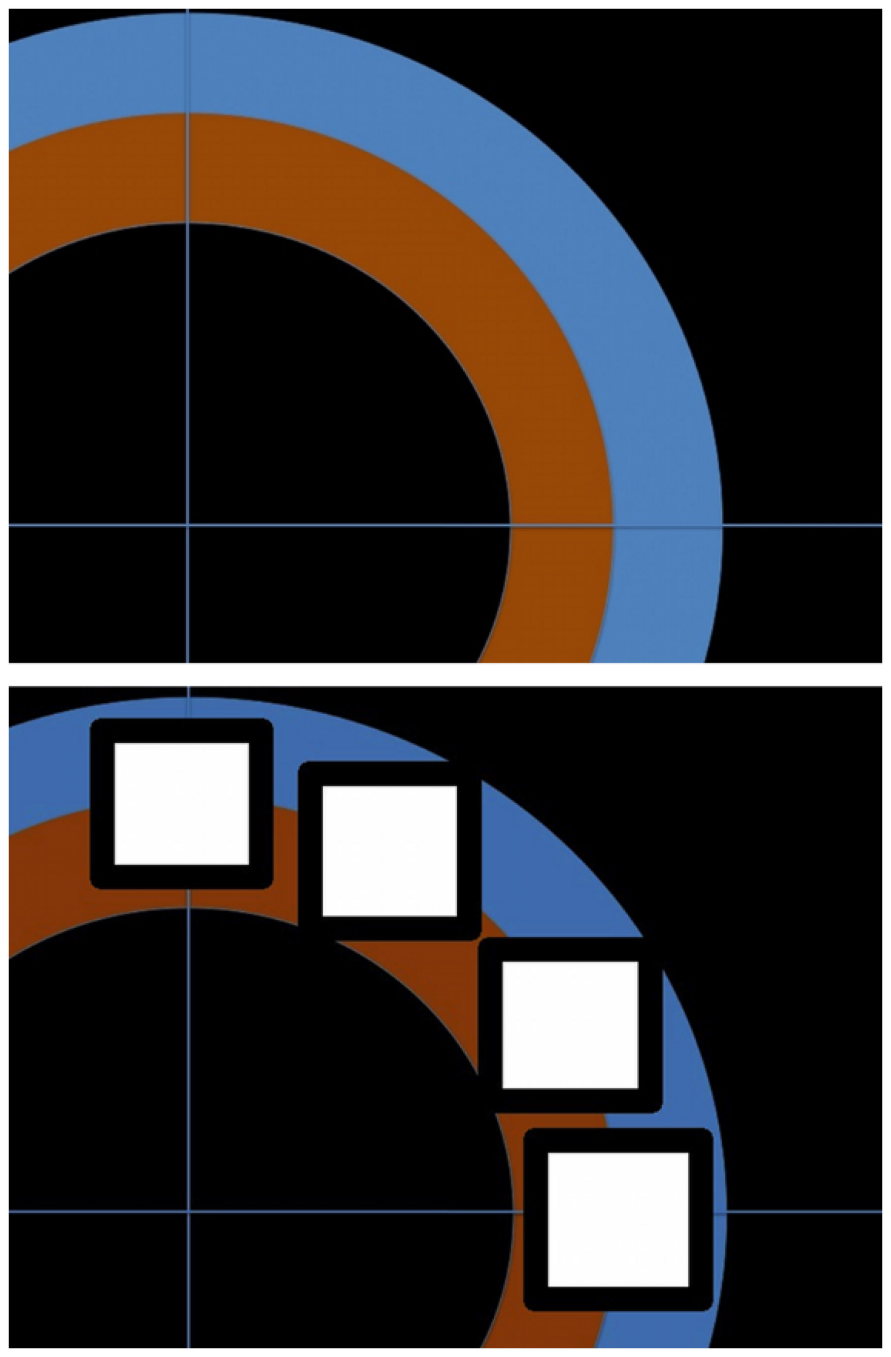
Touch-screen interfaces. Interfaces used for pre- and post-tests (top) and training session tests (bottom).

Both azimuth and elevation experiments started with a pre-training session test. In this pre-test, the speech and noise stimuli were repeated ten times, with virtual source positions chosen pseudo-randomly in the mentioned ranges. Participants were asked to point the perceived source position on a touch screen (see Figure 1 - top), on a continuum from front to right in the azimuth experiment, and from front to top in the elevation experiment. The vertical and horizontal axes in Figure 1 represented, respectively, the “front” (0°) and “right” (90°) directions in the azimuth experiment and the “top” (90°) and “front” (0°) directions in the elevation experiment. While pointing in that continuum, participants were asked to also report externalization levels. They were told that they should respond in the orange area if they felt the sound more inside the head, and in the blue area if the sound was felt outside the head. They were told to respond in a continuum, inner border being the most inside the head and outer border being 1 m away from the head. Each trial lasted for 3 s, with a 2 s interval between stimuli.

The participants then engaged in a training session in which the virtual source was restricted to four angular positions (in azimuth or elevation, depending on the experiment): 0°, 30°, 60° and 90° (represented by the white areas in Figure 1 - bottom). In each experiment a part of the participants trained with noise sounds, and the other part trained with speech sounds. 

The training followed the same steps as described in our previous work [13]:

1 Active Learning: The participants were informed they had five minutes to learn to identify source position and would be tested afterwards. While training, they were allowed to choose among the four source positions at will, by pointing at the corresponding white area.
2 Passive Feedback: 3 s sounds positioned randomly at one of the four possible options were played with an inter-stimulus interval of 4 s. In each trial, the participants had to identify source position by pointing the corresponding white area on the touch screen. The correct answer was shown immediately after each trial. The sounds were organized in sequences of 10. This training stage continued until the number of correct answers reached 80% (azimuth localization) or 70% (elevation localization) for two consecutive sequences,.

Post-training tests were then carried out using exactly the same stimuli and procedure of the pre-test. In the main condition, there were five post-tests: 1) immediately after training; 2) one hour later; 3) one day later, 4) one week later and 5) one month later. In the control condition, there were only two post-tests: 1) immediately after training and 2) either one day, one week or one month later (i.e. skipping one, two or three intermediate tests, respectively).

All participants took part in both the elevation and azimuth experiments, but both experiment type and trained stimulus type were counterbalanced. Therefore, part of the participants trained elevation first and the other part trained azimuth first. Part were trained in speech first and part were trained in noise first. Subjects trained in azimuth using speech were trained in elevation using noise; conversely, subjects trained in azimuth using noise were trained in elevation using speech. There were therefore 4 possible training orders. Here is an example of test sequence in the main condition, for a subject who trained elevation and speech first: 1) pre-test in elevation; 2) elevation training with speech sounds; 3) post-test in elevation; 4) pre-test in azimuth; 5) azimuth training with noise sounds; 6) post-test in azimuth; 7) one hour later, post-test in elevation immediately followed by post-test in azimuth; 8,9,10) similar post-tests a day, week, and one month later.

In the main condition, three subjects trained elevation and speech first, two trained azimuth and speech first, two trained elevation and noise first and two trained azimuth and noise first. Therefore, half participants started by training speech, half by training noise; half started by training elevation and half by azimuth. In the control condition, five subjects trained elevation first and four trained azimuth first; five trained speech first and four trained noise first (see Supporting Information S1 for a map of participant distribution across conditions).

All experiments took place in a quiet room with black walls and lights off. 

## Main Condition Results

This section presents the results of the azimuth and elevation experiments. The results are presented under different perspectives, resulting in five sub-sections: *Training Effect* analyses the effect of training on localization accuracy, and the influence of stimulus types on it. *Experience Effect* looks into the influence of prior elevation training in azimuth localization results and vice-versa. *Stimulus Position* analyses localization performance as a function of source azimuth/elevation. The *Time Effect* part observes the evolution of localization performance along time, based on post-test data. Finally, *Externalization* analyses the influence of time and stimulus type on reported externalization levels.

Results are expressed in localization error and externalization level. Localization error was computed as the average Euclidean distance between the position of each stimulus and the position of each corresponding response. It was first computed trial by trial, in degree, and then averaged according to the following variables to be analysed: by participant (data not shown), by group, by experiment, by stimulus type, by stimulus position, by test-session. Externalization levels were computed from the participant’s responses in a continuum between two colored areas, where the inner area of the arc corresponded to sounds perceived most inside the head, and the outer area of the arc corresponded to sounds perceived most outside the head. Pixel outputs were converted to a linear externalization scale, where value 0 then represented a response at the inner border of the arc, and value 100 corresponded to a response at the outer border of the arc. Value 50 was defined at the color border; this value represented the line between sounds perceived inside and outside the head.

### Azimuth experiment

#### Training effect

All ten participants took less than 20 minutes to reach the target localization accuracy. Four of them started the passive feedback phase already at 80 percent accuracy and only three took longer than 5 minutes. For those three subjects, a decrease in accuracy was found over the first trials of the passive feedback phase. 

**Figure 2:**
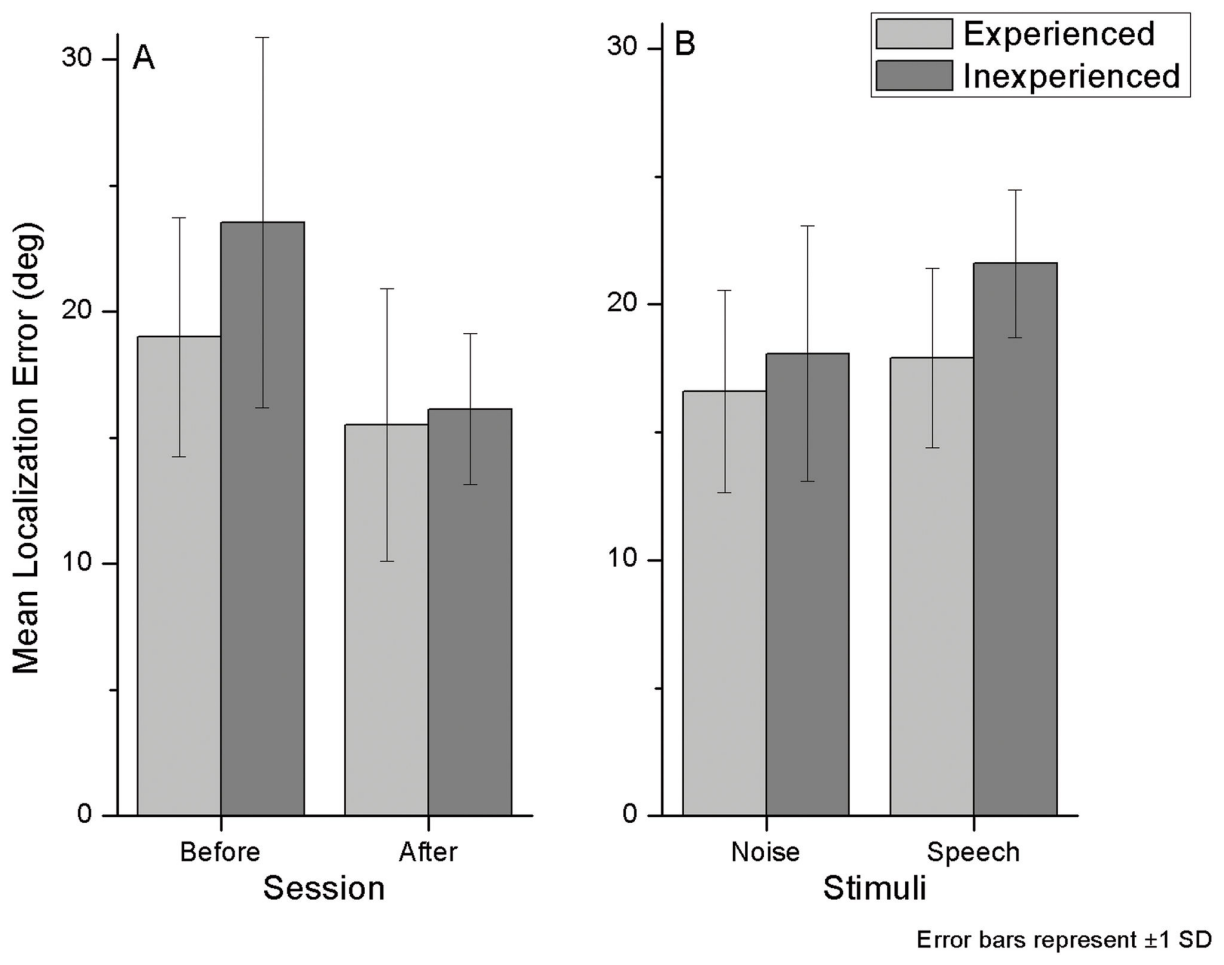
Azimuth localization mean error. Mean error as a function of training (A) and stimulus type (B).

All subjects improved localization with training. Figure 2 (left) shows mean localization errors before and after training. 

In the pre-test there was a mean localization error of 21.3°, while in the first post-test the mean error was reduced to 15.8°. This difference is statistically significant in a t-test (t_9_=5.4, p≤0.001). 

Those who trained with white noise sounds achieved higher accuracy levels than those who trained with speech. Immediately after training, the mean error in azimuth for those who trained with speech was 17.9°, while for those who trained with noise it was 13.8°. Those who trained with speech decreased on average 5.5° in speech localization error. Interestingly, they also improved in noise localization, with a 7.0° reduction in error. In a similar way, those who were trained with white noise decreased 4.7° in noise localization error, but they also decreased 4.6° in speech error. Localization was significantly better for both stimulus types after training (speech: t_9_=6.4, p≤0.001; noise: t_9_=2.56, p≤0.05).

#### Experience effect

Half of the participants took part in the elevation experiment first and, for this reason, were regarded as experienced when they started the azimuth experiment. Conversely, those who started by the azimuth experiment were considered experienced in the elevation experiment. 

Experienced participants indeed performed differently (see Figure 2). They started with an error level of 19.0°, against 23.5° in the inexperienced group. After training, there was still a small benefit, where experienced listeners had an error of 15.1° and the inexperienced listeners had an error of 15.5°, but this difference was not significant. Considering the localization error prior to training both in the azimuth and in the elevation experiment, there was a significant difference between experienced and inexperienced groups (t_9_=13.21, p≤0.001). 

Both experienced and inexperienced subjects had lower localization errors in noise stimuli than in speech stimuli. The experienced group had on average 16.6° of error for noise and 17.9° for speech sounds. The inexperienced group had 18.1° for noise and 21.6° for speech. These results indicate better localization accuracy for experienced listeners.

#### Time effect

**Figure 3:**
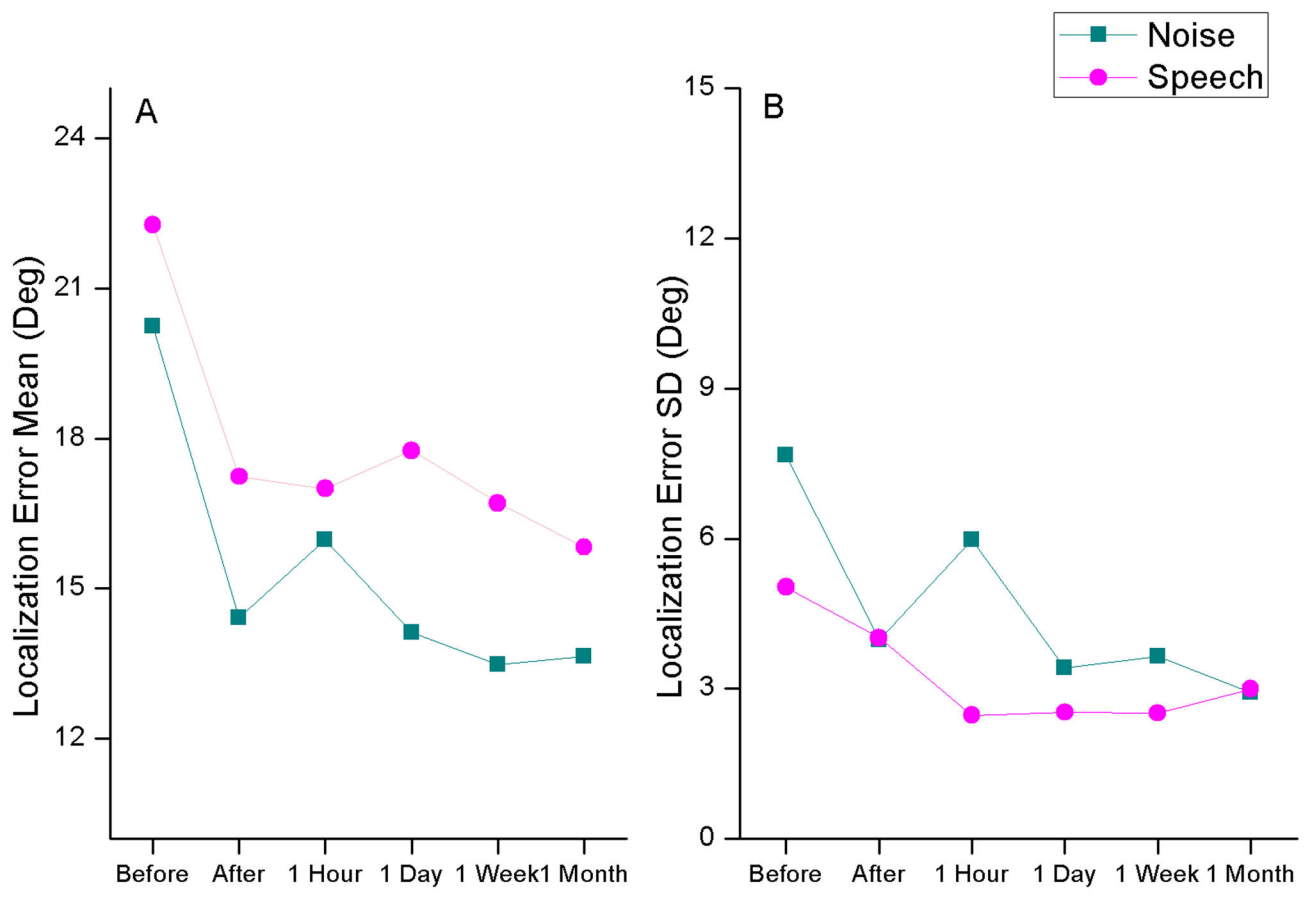
Average azimuth localization error with noise and speech stimuli along all experimental sessions (A) and respective standard deviation (SD) values (B).

Overall, there was a clear training benefit with persistent effects over time. Before training, there was an average localization error of 21.3°. Immediately after training, the error was reduced to 15.8°, and then it remained stable at the subsequent tests, with 16.5°, 16.0°, 15.1° and 14.7° values one hour, one day, one week, and one month later, respectively. Along time, localization errors were persistently lower for noise stimuli than for speech stimuli, as depicted in Figure 3. 

In a factorial ANOVA, analyzing the effects of stimulus type and test session, these differences were confirmed. The main effect of test session was found to be significant (*F*_*1,5*_=6.42, p≤0.001). In a post-hoc Sheffé analysis, it was found that such differences were only significant between the pre-test session and all others. None of the post-training test sessions differed significantly from the others. The main effect of stimulus type was also significant (*F*_*1,5*_=10.45, p≤0.005), revealing that the benefit of noise over speech as stimuli in the localization task was consistent along time.

#### Stimulus position

Localization accuracy varied widely with stimulus position. There was also a large variability among subjects. The largest localization errors were found for the 10°-40° azimuth range, where results before training were not statistically different from response at chance. The best accuracy was found for frontal and lateral stimuli. As an example, the average localization error for 0° azimuth was 21.2° before training and 10.98° after training. This difference is statistically significant in a t-test (t_9_=2.8, p≤0.05). On the other hand, localization error at azimuth 60°, also a trained position, improved from 12.0° to 10.0°, a difference which was not statistically significant. The stimulus positions for which localization improved the most with training were 0°, 10° and 20°, with 10.2°, 8.0° and 5.9° absolute mean error reductions, respectively. The positions for which localization improved the least with training were 60°, 50° and 90°, with mean error lowered by 2.0°, 3.0° and 3.2°, respectively. Result differences were statistically significant for all source positions between 0° and 50° (inclusive). Training reduced localization error for all azimuths, including untrained ones, and the magnitude of error reduction was not related to direct training. Therefore, the training effect was not limited to the trained positions, and many untrained positions improved more than trained ones.

#### Externalization

**Figure 4:**
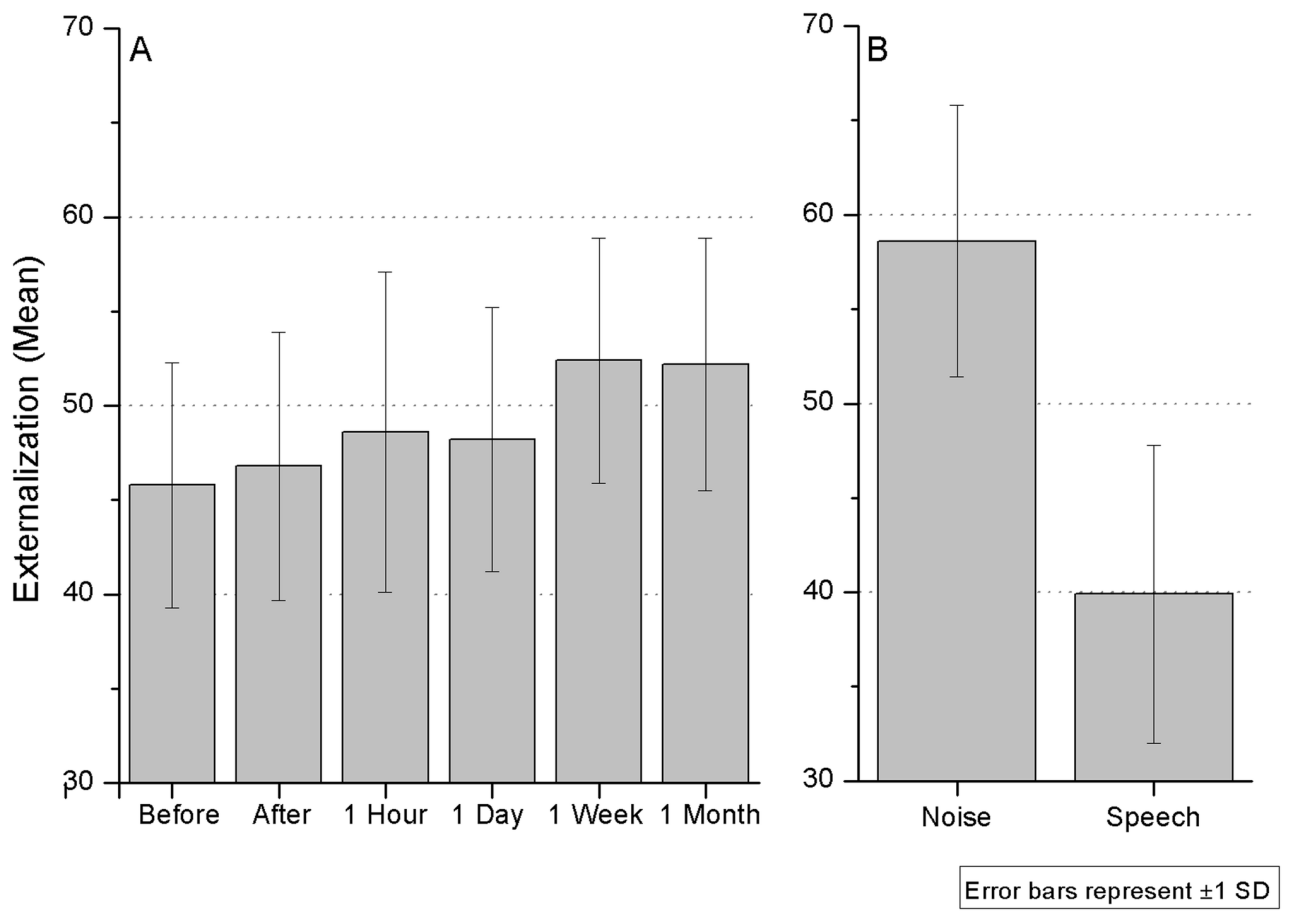
Externalization levels across time (A) and by stimulus type (B). Level of 50 represents the threshold between inside the head (values under 50) and outside the head.

Overall, externalization levels were low. On average, sounds were perceived mostly inside the head in the first and second day (see Figure 4). There was a mean externalization of 46 before training, 47 after training, 48 one hour later and 48 one day later. But one week and one month later mean externalization was 52. This might indicate a tendency for greater externalization as listeners become acquainted with the localization cues provided by non-individualized HRTF sounds, but evolving at a slower pace than localization accuracy. In paired-sample t-tests, results showed that only the first and last sessions were statistically different from each other (t_9_=-2.8, p≤0.01).

Externalization seemed to depend strongly on stimulus type. The global externalization level for the noise sounds was 59, against 40 for speech sounds, which indicates that noise sounds were mostly externalized, while speech was more often perceived inside the head. This effect was significant in a t-test (t_9_=2.1, p≤0.05).

### Elevation experiment

#### Training effect

The outcomes of the elevation experiment were very similar to those of the azimuth experiment. All participants improved with training and all training sessions lasted less than 20 minutes. In the pre-test, the mean localization error was 29.3°, higher than that for azimuth. Considering that a subject answering randomly would obtain a mean localization error of 33°, this value was also close to random levels. In the post-test, the error was 25.9°. This difference is significant in a t-test (t_9_=3.39, p≤0.01). Those who trained with speech sounds improved accuracy by an average of 3.4°, while those who trained with noise improved by 5°. Those who trained with noise improved in localizing speech and vice versa, but the effect was not as strong as in the azimuth case. Those who trained speech improved 6.7° in speech localization, and only 3.2° in noise localization. Those who trained noise improved 4.9° in noise and only 2° in speech. On average, both speech and noise sounds were localised more accurately after training, and those differences are statistically significant (speech: t_9_=2.83, p≤0.05; noise: t_9_=4.3, p≤0.005).

#### Experience effect

**Figure 5:**
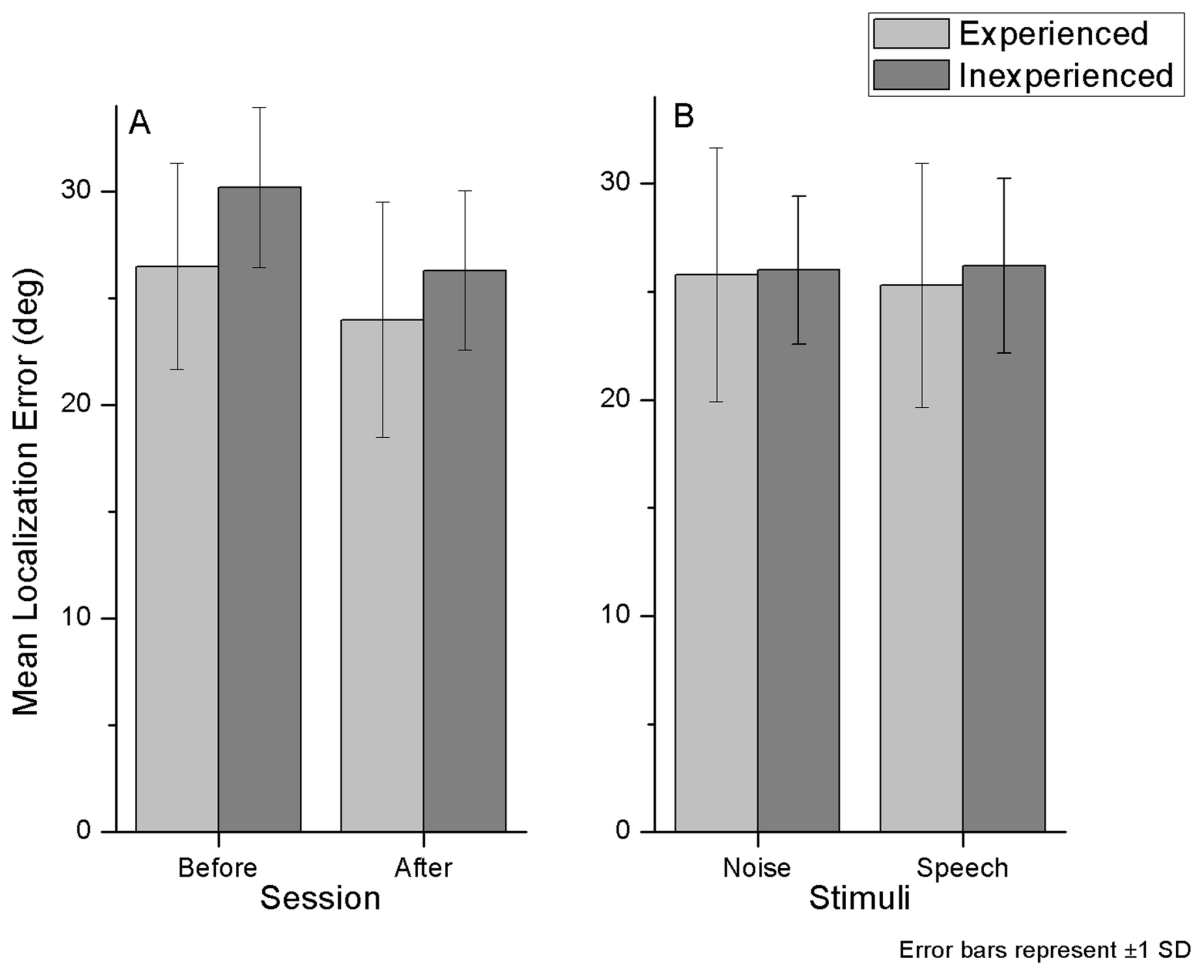
Elevation localization mean error. Mean error as a function of experience (A) and stimulus type (B).

As previously analyzed in section 3.1.2, experienced subjects performed statistically better in the pre-test than inexperienced subjects. The mean error for experienced listeners prior to training was 26.5°, while for inexperienced listeners it was 30.2°. Again, this benefit was still found after training, where inexperienced subjects had a mean error of 26.3° against 24° of the experienced group. There were, however, no accuracy differences by both trained and untrained subjects in the localization of speech and noise sounds prior to elevation training (Figure 5).

#### Time effect

The general tendency over time was similar to that observed in the azimuth experiment, with a clear improvement in localization with training and a stabilization after that. Before training, the average localization error was 29.3°. Immediately after training, the average error was reduced to 25.2° and stayed at 25.4°, 24.8°, 25.4° and 24.8° in the subsequent test sessions. There were, however, no relevant differences between noise and speech sound. On average, the error was 25.7° for speech and 25.9° for noise.

In a factorial ANOVA for stimulus type and test session effects on error levels, no significant stimulus type effect was found (F_*1*_=1.01, n.s.). There was, however, a significant effect of test session (F_*5*_=3.28, p≤0.01).

#### Stimulus position

**Figure 6:**
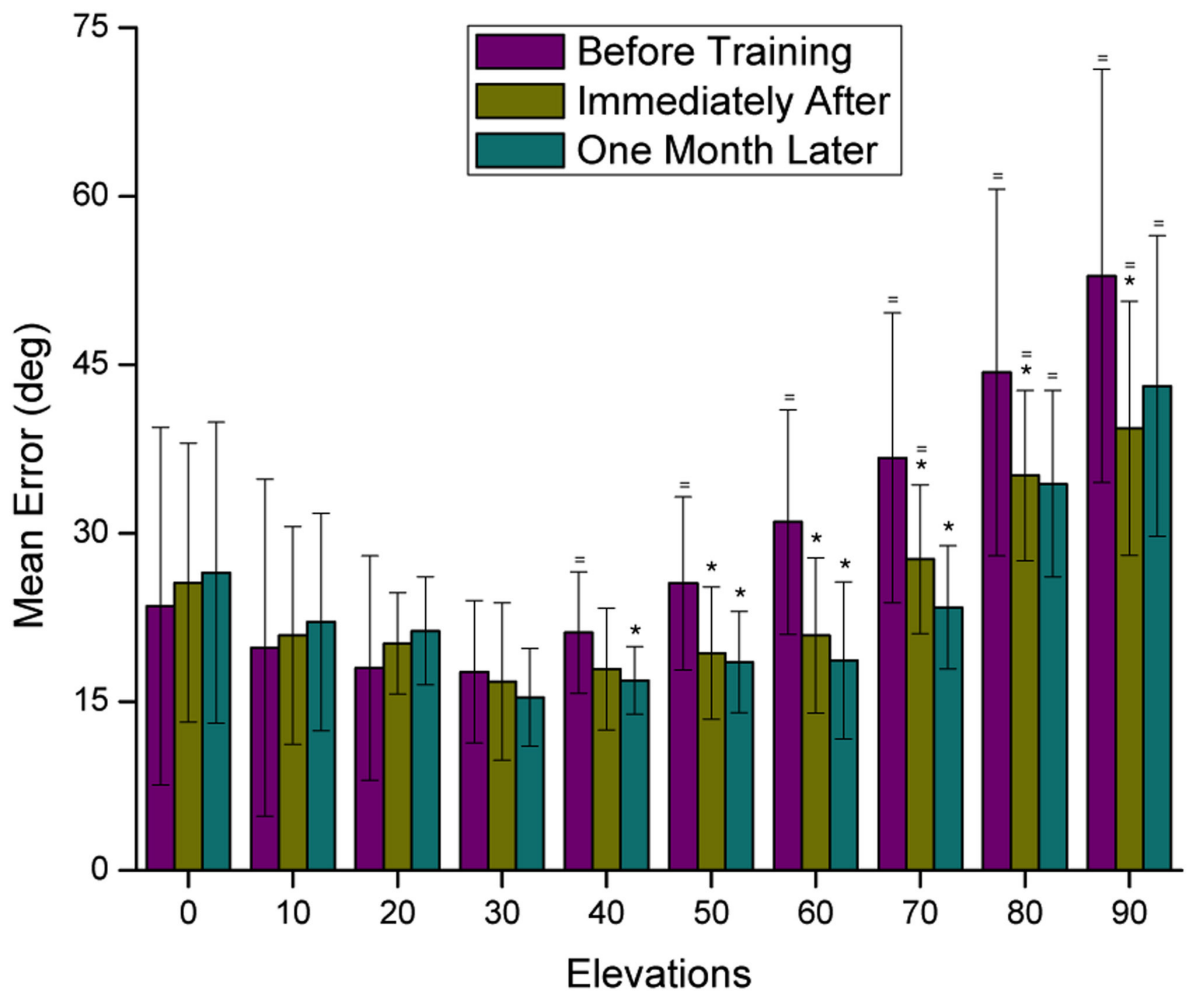
Mean error (deg) in localization of sounds across elevation, before, immediately after, and one month after training. Error bars represent ± one standard deviation. The symbol = above bars represents values that are not statistically different from the response at random for that given position, in a t-test for mean difference. The symbol * represents statistically significant differences between the pre- and post-test in a paired sample t-test.

As depicted in Figure 6, the error of sound position estimation varied across elevation. The largest localization errors prior to training were at higher elevations, namely at 70°, 80° and 90°, with 36.7°, 44.3° and 59.9° mean errors, respectively. All elevation estimates from 40° to 90° were not different from random responses before training. Conversely, the smallest localization errors prior to training were at lower elevations, from 0° to 40°. 

With training, most stimuli elevations were better localized. There were statistically significant localization improvements in elevations comprised between 40° and 90°. Interestingly, these were the same elevations that were at chance level prior to training.

However, at three stimulus positions (0°, 10° and 20°), no improvement was observed with training. It should be noticed that these stimuli were already localized above chance before training.

In a similar way to what was observed in the azimuth experiment, the localization of some trained source positions did not improve or improved less than some untrained positions. This was the case of the 0° and 30° sounds, which were trained but were not better localized in the post-tests. On the other hand, there were several untrained source positions for which localization accuracy improved significantly, namely the 40°, 50°, 70° and 80°. These stimuli were among those with the largest localization improvement after training. The largest accuracy improvement, however, was observed for the 90° sounds, where after training there was a 13.5° error reduction and one month later there was still a 9.8° reduction.

#### Externalization

**Figure 7:**
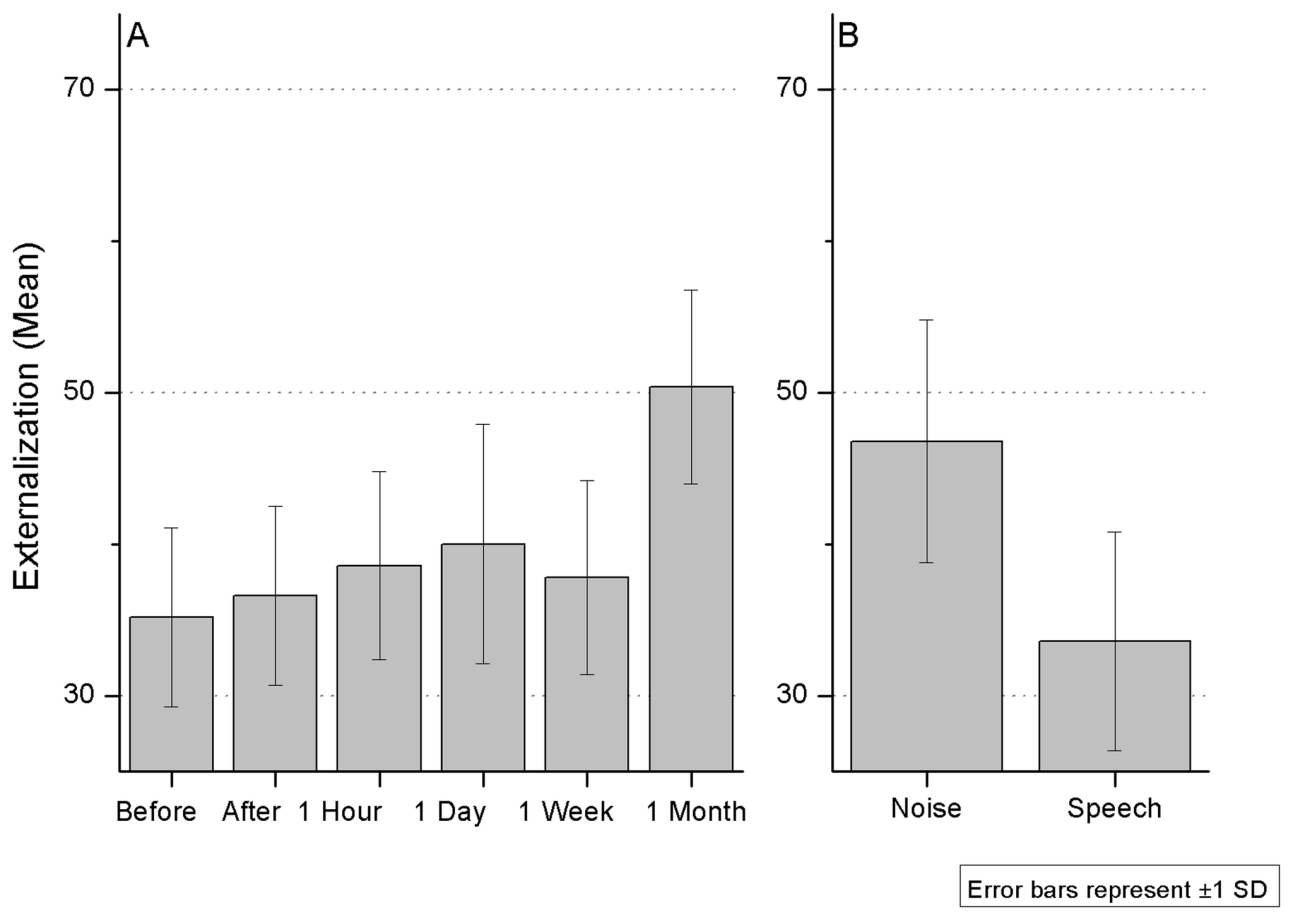
Externalization levels in the elevation experiment across test sessions (A) and by stimulus type (B).

Externalization levels in the elevation experiment were even lower than in the azimuth experiment. Taking 50 as the threshold, externalization levels compatible with outside-the-head sound perception only appeared in the last session (Figure 7). In the first session, sounds were mostly perceived inside the head (35.2) and values stayed quite stable along the first 4 post-tests, despite a small value increase: 36.6 immediately after and 38.6, 40 and 37.8 respectively one hour, one day and one week later. However, the one month post-test showed an externalization level of 50.4. Indeed, only the pre-test and the one month post-test results differed significantly in a t-test analysis (t_9_= -2.8, p≤0.01). 

Comparing stimulus types, noise again yielded more outside-the-head estimations, although the mean values were still under the cut point (46.8). Like in the azimuth experiment, the speech sounds remained mostly inside the head, with very low externalization levels (33.6). The difference in externalization between noise and speech sounds was statistically significant (t_9_=1.33, p≤0.01). 

## Control Condition Results

The control condition experiments were conducted to assess the impact of all successive retesting sessions on the long-term results. The effect of successive information retrieval on memory is well known; information that is used more often is less likely to be forgotten. Here, it was hypothesized that the reason that listeners localized so accurately and had such good externalization levels one month after the short training session was because of successive retrieval of the trained information.

Here we conducted a new set of experiments in azimuth and elevation learning, where all procedures were kept the same except for the post-test sessions. There were three experimental groups: the one-day group, that was only retested one day after training; the one-week group, that was tested one week later; and the one-month group, only retested one month after training. Therefore there were always a total of three test sessions per group: the pre-test, the post-test immediately after training, and the final post-test that varied across groups.

**Figure 8:**
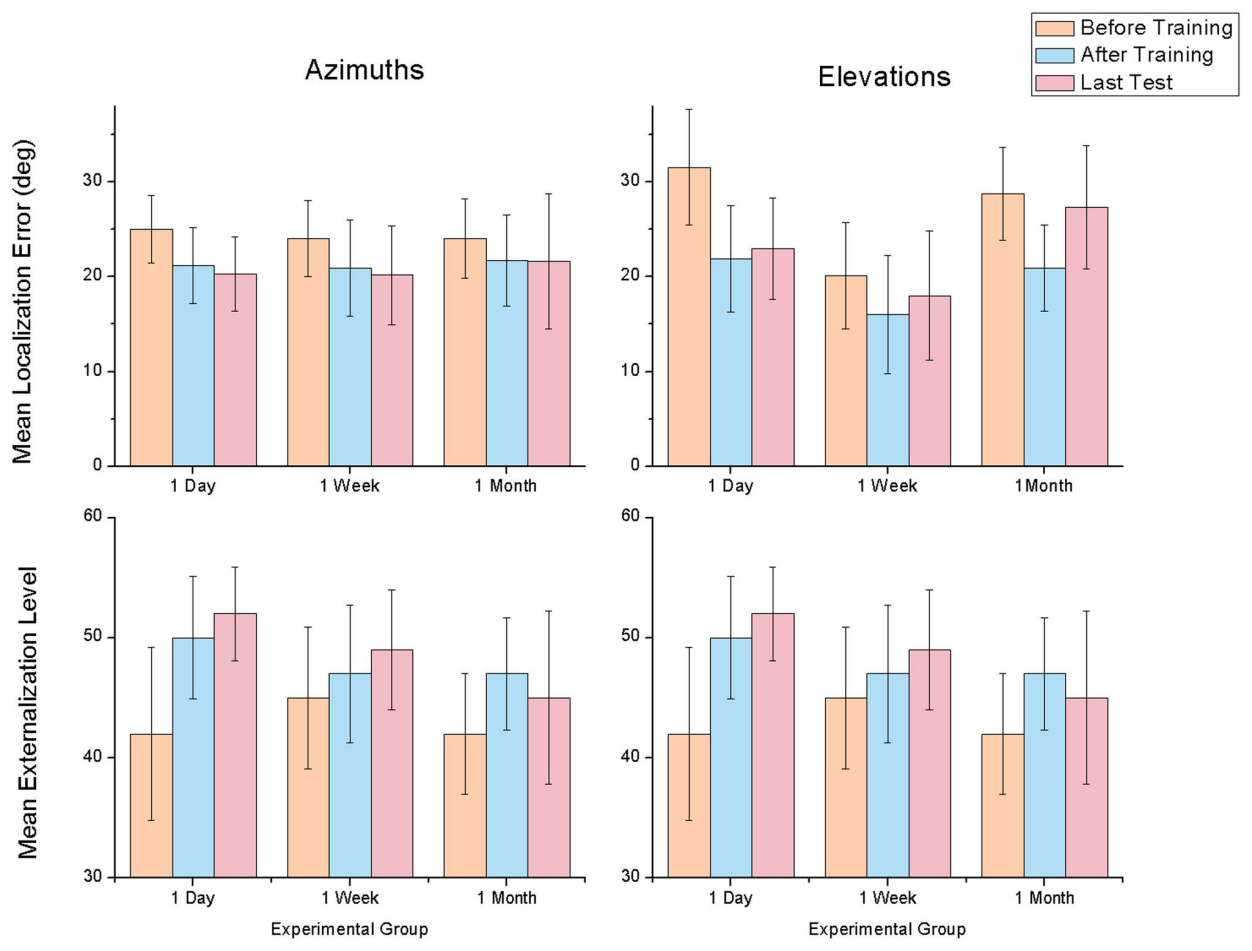
Results of the control condition. Top graphics show localization error and bottom graphics display externalization levels. Left shows azimuth results and right shows elevation results. Results are organized according to the three experimental groups: one-day group was only retested one day after training; one-week group was retested only one week after; one-month was retested one month later. Bars represent results before training, after training, and in the last post-test (a day, week or month later). Error bars represent ±1 SD.

The results of these experiments are summarized in Figure 8.

Both in the azimuth and in the elevation experiments, the average localization error decreased after training, but there were different long-term effects of such training. In azimuths, the average error reduction was of 3.1° immediately after training. One day later, there was an error reduction of 4.7°, while one week later it was 3.9° and one month later it was 2.4°. In elevations there was on average an improvement of 7.2° in localization accuracy immediately after training. However, in the long term there were differences between groups. The one-day group had an error reduction of 8.6° comparing to the pre-test. The one-week had only a benefit of 2.1°, and the one-month group only had a benefit of 1.4°. Unfortunately, since there were only three participants per group, there was not enough sample size to conduct meaningful statistical tests.

Regarding externalization there were also different effects between the azimuth and the elevation experiment. In azimuth, all groups increased externalization after training, but with different patterns. The one-day group started with an average externalization level of 42 and increased to 50 immediately after training and to 52 the next day. The one-week group also increased steadily in azimuth externalization. They started with a level of 45 and reached 47 after training and 49 one week later. In the one-month group the results were not as clear. They started with an average of 42, achieved 47 after training and then reduced back to 45 one month later. In elevation, externalization effects were even smaller. The one-day group had a small externalization evolution, from 43 in the pre-test to 45 after training and 47 one day later. The one-week group failed to reveal any long-term externalization effects, with a level of 43 before training, 49 immediately after training, but again 42 one week later. A similar effect was observed in the one-month group: it started with a 45 externalization level and increased to 49 after training, but was reduced to 43 one month later. Such results might reveal the relevance of successive contact with the new head related cues, namely for the learning of sound elevation.

## Discussion

The general scope of this work was to analyze the generalizations in learning auditory localization with altered head-related sound cues along time. It was expected that such analysis would cast new insights on how the auditory spatial maps are formed and recalibrated, how the brain associates the sound cues to localizations in space, and how this association is represented. For that purpose, subjects were trained to localize speech or noise sounds in azimuth and in elevation and retested at several time intervals until one month later. We then analyzed the evolution of localization errors and externalization levels along time.

All subjects improved with training both in azimuth and in elevation. Some benefit was found for white noise sounds over speech. In azimuth, both experienced and inexperienced listeners had lower localization errors in noise than in speech. These sounds were also consistently more externalized than speech sounds. Such results are easily explained by the fact that wideband noises carry much more spectral information, and therefore more cues, for the learning/retrieving process. Although such sounds are not as natural as human speech, they might be preferable for training purposes and even to provide more out-of-the-head experiences.

An unexpected effect of previous experience was also found. Both in elevation and in azimuth, listeners localized significantly better prior to training if they had been previously trained in the other task. This result points to a first interpretation standing from this study: the neural representation of the new head related localization cues might take the form of a generic congruent model, not a specific cue-to-location pair. As such, totally untrained elements from the same head-related model might still benefit from training.

This interpretation is consistent with the results regarding the stimulus position. Indeed, both in the azimuth and in the elevation experiments only four sound positions were trained. Nevertheless, error reduction was found in many other stimuli positions. Also, some untrained sounds positions had higher error reductions than some trained ones. This result, congruent with our previous findings [13], is consistent with the interpretation that a global new head model is formed as subjects learn to localize with the new cues. Localization errors were also varied in continuums, namely after training: in azimuth, they were higher in the intermediate area between front and lateral positions; in elevation they were better in higher positions and gradually became worse as they became more frontal. This might outline a global congruent representation of the new sound cues, which is formed from some learned features and then mentally interpolated in a consistent map. Shinn-Cunningham [15] proposed a similar explanation for the adaptation to remapped auditory localization cues. She proposed that subjects cannot adapt to nonlinear rearrangements of localization cues. In spatial adaptation subjects would learn to interpret a continuous internal decision variable differently than normal; they do not learn to associate discrete stimulus/response pairs.

The analysis of results along time also revealed some interesting effects. The learning of the new head-related cues had an enduring effect over time, both in azimuth and in elevation. The benefits were still found one month after the short training session. The fact that this new head-related map is remembered for longer periods of time is consistent with previous data on auditory remapping [8]. In that work, subjects wore ear molds for nineteen days. By the end of such time they had learned the new auditory map and were able to localize sounds in elevation, but they were still as accurate as prior to training in localizing with their own ears. Here subjects continued wearing and localizing with their own ears, but kept the representation of the new map for a period of one month. It is therefore arguable that listeners can learn and use simultaneous head-related maps, much like learning and using simultaneous languages. But in light of the control condition results it is arguable that some additional contact with the learned cues helps maintain the new maps. This effect was not the same for both azimuth and elevation. Azimuth space was sustainably maintained up to one month after training without any additional session, but elevation was gradually more affected with longer periods between training and post-testing. This outlines that possibly the new auditory map represents binaural and monaural cues differently. Indeed, the differences between azimuth and elevation processing in the brain are related to the most prominent use of binaural cross correlation for azimuth and of spectral cues for elevation. These results might reveal different long-term encoding of both types of signal. 

The analysis of the externalization levels brings further evidence at this level. Sounds were overall poorly externalized, but such externalization improved with time. It took longer to reach the external threshold for elevation than for azimuth, again representing some differences in how the brain deals with both cue types. Interestingly, although externalization followed a similar evolution as localization accuracy, both improving with time and being better achieved with the white noise stimuli, it showed more cumulative effects. As such, although localization results tended to stabilize with time, externalization levels tended to improve with the successive tests. In the control condition it was noticeable that the closer together the test sessions, the higher the impact on enhanced externalization. It becomes therefore arguable that externalization might be achieved with greater cue familiarity, or, in the language analogy, it might be associated with greater fluency or naturalness in using the second head-related spatial map.

Finally, some issues regarding the training method and what was effectively learned should be addressed. Did subjects learn new head-related cues, or were they merely learning sound features or cues? Firstly, it should be restated that trained and tested stimuli were not the same. For each experiment, subjects were trained in only one sound type, at only 4 positions. They revealed benefits in another sound type and in many other sound positions. So we can conclude that subjects were not simply finding cues in the sounds when learning them. There was also benefit just by participating in a previous experiment. But from azimuth to elevation, only the 0° was the same. No sound feature could be learned in all the other sounds.

But were they learning the task? Indeed all participants were inexperienced. It can be argued that benefits on localization were simply due to adjusting to the task and response interface. We don’t have enough information about those processes to offer a conclusive response. But such adaptation should happen within the first trials of the experiment and reach a plateau very fast. In most psychophysical research, an extremely short familiarization with experiments, with only a handful of trials, is usually enough to discard such effects. In this study, since we intended to avoid any previous experience, no such experiment was run. In any case, in our previous work with similar stimuli [13], there was such a control experiment. No benefit was found from prolonged exposure to the task. Performance in localization was constant throughout ten experimental blocks of localization without feedback or training. So task learning might not have played a relevant role in our current findings. As a final remark, the training sessions presented totally different task and different response interfaces from the test-sessions. So it can be stated that subjects were not trained in the task during the training session. It was, however, precisely after the training sessions that the largest improvements were found.

Here we argue that the improvement found after training was mostly due to an adaptation to new, non-individualized HRTF. We argue this based on previous evidence that training to localize with one’s own HRTF leads to little or no improvements, whereas localization accuracy with altered HRTFs strongly benefits from training [10].

In any case, additional experiments should be run, with alternative tasks or learning methods, to better understand the underlying mechanisms of the data presented here. It would be particularly interesting to compare these results to more implicit learning tasks, where subjects would not be aware of the purposes of the experiment. There are indeed other approaches to training, with more implicit learning, like audiovisual settings with motion or real-time systems allowing for visual or vestibular feedback [8,9,11,12]. However, such paradigms are very different in nature, often involving multisensory processes, and are therefore hard to compare. 

## Conclusion

This study brought new insights on the learning and generalization of auditory space. We found evidence that a brief training session with only a selection of sounds leads to a general improvement in the localization of trained and untrained sounds in trained and untrained positions. These effects are still observed one month after training. Externalization levels are also increased by training, although not directly related to localization accuracy levels. Information retrieval might affect the long-term effects of training, namely for localization in elevation. We conclude that sound localization with altered cues is easily trained and subject to generalization effects across sound type, space and time.

## Supporting Information

Supporting information S1 **Subject distribution across experimental conditions.**
(PDF)
