# Supplementary material for: Learning Auditory Space: Generalization and Long-Term Effects
Source: PLoS One. 2013 Oct 22;8(10):e77900. doi: 10.1371/journal.pone.0077900 (PMC3805533; doi:10.1371/journal.pone.0077900)
Supplement: Supporting information S1 — Subject distribution across experimental conditions. (PDF) [file pone.0077900.s001.pdf]

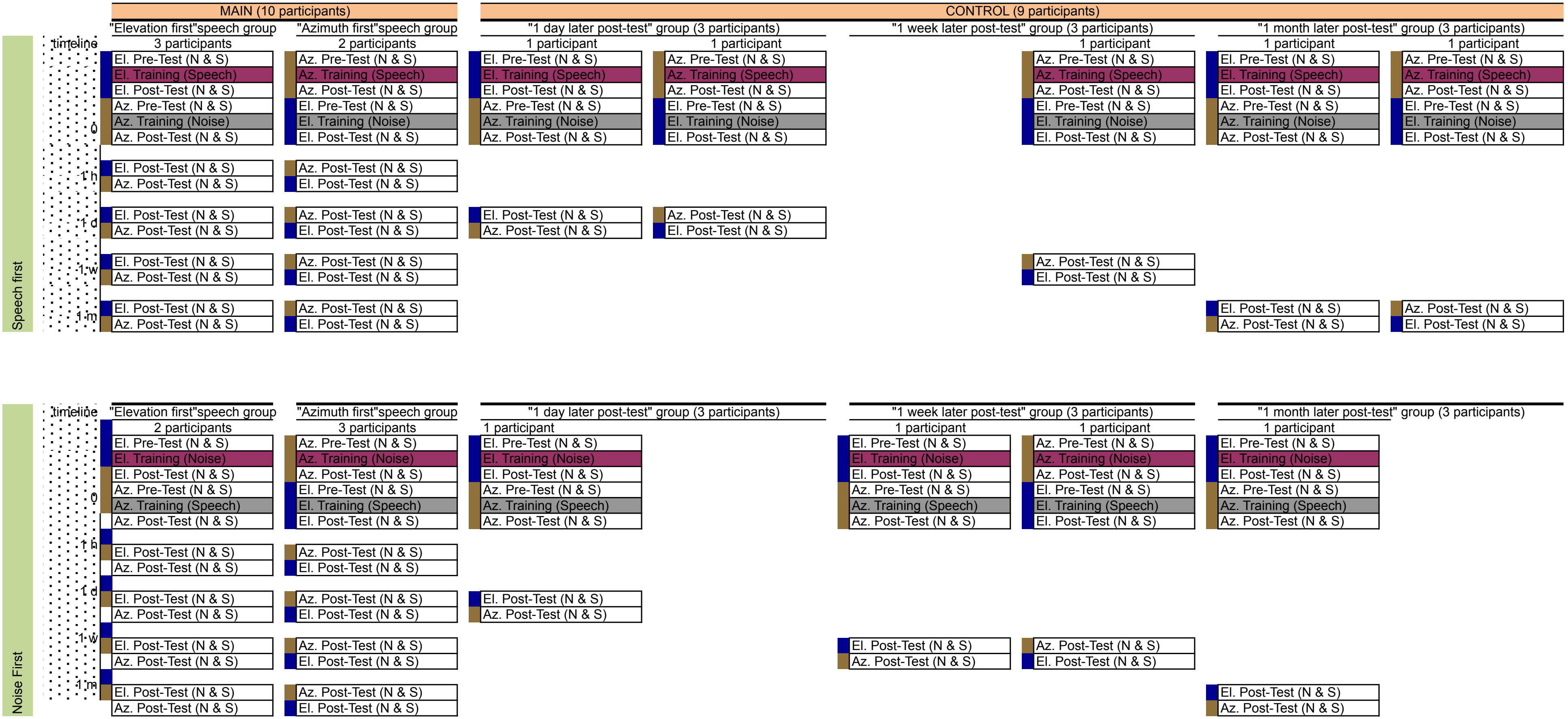
MAIN (10 participants)
CONTROL (9 participants)
Speech first
timeline
0
1 h
1 d
1 w
1 m
"Elevation first"speech group
3 participants
El. Pre-Test (N & S)
El. Training (Speech)
El. Post-Test (N & S)
Az. Pre-Test (N & S)
Az. Training (Noise)
Az. Post-Test (N & S)
El. Post-Test (N & S)
Az. Post-Test (N & S)
"Azimuth first"speech group
2 participants
Az. Pre-Test (N & S)
Az. Training (Speech)
Az. Post-Test (N & S)
El. Pre-Test (N & S)
El. Training (Noise)
El. Post-Test (N & S)
Az. Post-Test (N & S)
El. Post-Test (N & S)
"1 day later post-test" group (3 participants)
1 participant
El. Pre-Test (N & S)
El. Training (Speech)
El. Post-Test (N & S)
Az. Pre-Test (N & S)
Az. Training (Noise)
Az. Post-Test (N & S)
El. Post-Test (N & S)
Az. Post-Test (N & S)
1 participant
Az. Pre-Test (N & S)
Az. Training (Speech)
Az. Post-Test (N & S)
El. Pre-Test (N & S)
El. Training (Noise)
El. Post-Test (N & S)
Az. Post-Test (N & S)
El. Post-Test (N & S)
"1 week later post-test" group (3 participants)
1 participant
Az. Pre-Test (N & S)
Az. Training (Speech)
Az. Post-Test (N & S)
El. Pre-Test (N & S)
El. Training (Noise)
El. Post-Test (N & S)
Az. Post-Test (N & S)
El. Post-Test (N & S)
"1 month later post-test" group (3 participants)
1 participant
El. Pre-Test (N & S)
El. Training (Speech)
El. Post-Test (N & S)
Az. Pre-Test (N & S)
Az. Training (Noise)
Az. Post-Test (N & S)
El. Post-Test (N & S)
Az. Post-Test (N & S)
1 participant
Az. Pre-Test (N & S)
Az. Training (Speech)
Az. Post-Test (N & S)
El. Pre-Test (N & S)
El. Training (Noise)
El. Post-Test (N & S)
Az. Post-Test (N & S)
El. Post-Test (N & S)
Noise First
timeline
0
1 h
1 d
1 w
1 m
"Elevation first"speech group
2 participants
El. Pre-Test (N & S)
El. Training (Noise)
El. Post-Test (N & S)
Az. Pre-Test (N & S)
Az. Training (Speech)
Az. Post-Test (N & S)
El. Post-Test (N & S)
Az. Post-Test (N & S)
"Azimuth first"speech group
3 participants
Az. Pre-Test (N & S)
Az. Training (Noise)
Az. Post-Test (N & S)
El. Pre-Test (N & S)
El. Training (Speech)
El. Post-Test (N & S)
Az. Post-Test (N & S)
El. Post-Test (N & S)
"1 day later post-test" group (3 participants)
1 participant
El. Pre-Test (N & S)
El. Training (Noise)
El. Post-Test (N & S)
Az. Pre-Test (N & S)
Az. Training (Speech)
Az. Post-Test (N & S)
El. Post-Test (N & S)
Az. Post-Test (N & S)
"1 week later post-test" group (3 participants)
1 participant
El. Pre-Test (N & S)
El. Training (Noise)
El. Post-Test (N & S)
Az. Pre-Test (N & S)
Az. Training (Speech)
Az. Post-Test (N & S)
El. Post-Test (N & S)
Az. Post-Test (N & S)
1 participant
Az. Pre-Test (N & S)
Az. Training (Noise)
Az. Post-Test (N & S)
El. Pre-Test (N & S)
El. Training (Speech)
El. Post-Test (N & S)
Az. Post-Test (N & S)
El. Post-Test (N & S)
"1 month later post-test" group (3 participants)
1 participant
El. Pre-Test (N & S)
El. Training (Noise)
El. Post-Test (N & S)
Az. Pre-Test (N & S)
Az. Training (Speech)
Az. Post-Test (N & S)
El. Post-Test (N & S)
Az. Post-Test (N & S)
